# *Penium margaritaceum*: A Unicellular Model Organism for Studying Plant Cell Wall Architecture and Dynamics

**DOI:** 10.3390/plants3040543

**Published:** 2014-11-18

**Authors:** David S. Domozych

**Affiliations:** Department of Biology and Skidmore Microscopy Imaging Center, Skidmore College, Saratoga Springs, NY 12866, USA; E-Mail: ddomoz@skidmore.edu; Tel.: +1-518-580-5075; Fax: +1-518-580-5071

**Keywords:** *Penium*, cell wall, pectin, live cell labeling, monoclonal antibody, protoplast, transformed cell lines

## Abstract

*Penium margaritaceum* is a new and valuable unicellular model organism for studying plant cell wall structure and developmental dynamics. This charophyte has a cell wall composition remarkably similar to the primary cell wall of many higher plants and clearly-defined inclusive zones containing specific polymers. *Penium* has a simple cylindrical phenotype with a distinct region of focused wall synthesis. Specific polymers, particularly pectins, can be identified using monoclonal antibodies raised against polymers of higher plant cell walls. Immunofluorescence-based labeling is easily performed using live cells that subsequently can be returned to culture and monitored. This feature allows for rapid assessment of wall expansion rates and identification of multiple polymer types in the wall microarchitecture during the cell cycle. Cryofixation by means of spray freezing provides excellent transmission electron microscopy imaging of the cell, including its elaborate endomembrane and cytoskeletal systems, both integral to cell wall development. *Penium*’s fast growth rate allows for convenient microarray screening of various agents that alter wall biosynthesis and metabolism. Finally, recent successful development of transformed cell lines has allowed for non-invasive imaging of proteins in cells and for RNAi reverse genetics that can be used for cell wall biosynthesis studies.

## 1. Introduction

The utilization of new research methods and strategies emerging from technologies in molecular genetics, immuno-binding/cytochemical labeling, high resolution microscopy and spectroscopy have recently provided significant insight into deciphering the complexities of the plant cell wall [1,2,3,4,5,6]. The current, yet rapidly evolving, working model of the cell wall is one of a fibrous composite of polymers, primarily polysaccharides and glycoproteins, which are incorporated into specific loci of the wall and are subsequently modified at various intervals during the life of the plant cell [7,8,9,10]. Microfibrillar cellulose forms the load-bearing framework of the wall and is tethered by hemicelluloses into a stable network and embedded in a matrix of pectins. Structural proteins (e.g., extensin and arabinogalactan proteins), enzymes (e.g., pectin methyl esterase or PME, wall-associated kinases or WAKs), cations, polyphenolics, like lignins, and water also contribute to wall infrastructure. The components of the cell wall form specific structural domains around the plant cell that, in turn, are vital for such functions as controlled expansion and division, rigidity, defense and cell-cell adhesion, to name just a few. A significant portion of the plant cell’s genome is devoted to the biosynthesis and metabolism of its cell wall [11] that, in turn, is regulated by complex signal transduction cascades responding both to internal development prompts and environmental stresses [12,13,14]. Further elucidation of these cell wall-based phenomena will be critically important in many realms of basic and applied biology. This includes enhancing our knowledge of the living dynamics of a plant cell, the evolution of plants, especially over the last 500 million years of land plant evolution, and the ever increasing use of cell walls in the food, paper, building, textile, pharmaceutical and biofuels industries.

Much of what we have learned about the plant cell wall has been derived from studies of multicellular plants and, in particular, outstanding model organisms, like *Arabidopsis* *thaliana*. Over the past few decades, the emergence of highly detailed molecular data and protocols and their synthesis with experimental data derived from an easily-manipulatable organism, like *Arabidopsis*, has provided an efficacious system for resolving specific questions about the cell wall dynamics [15,16,17]. Likewise, the use of tissue culture-based systems, like tobacco cell suspension cultures, has aided in resolving the basics of the cell walls of individual cells [18]. However, these systems have some inherent limitations. For example, it is exceptionally difficult to resolve the cell wall structure and dynamics of a single cell situated in a plant organ/tissue composed of thousands of cells. Likewise, tissue culture-based cells are maintained under notably artificial conditions, and results derived from studies with them must be interpreted very carefully. Ideally, the utilization of a model plant that is naturally single celled, whose cell cycle can be synchronized (*i.e.*, cell wall developmental stages carefully controlled) and one that is easy to manipulate for experimental analyses would be ideal for dissecting the structure and function of the cell wall. The Charophycean green algae or charophytes (Streptophyta), the group of green algae most closely related and ancestral to land plants, may provide us with these models.

Recently, biochemical and immunobinding-based screening (including, comprehensive polymer profiling, CoMPP, and immunocytochemistry) has revealed that the charophytes have cell wall polymer composition profiles that are remarkably similar to those of land plants [19,20,21]. This includes a cellulose microfibril infrastructure with associated hemicelluloses (mannans, xylans, xyloglucans) embedded in a pectin matrix. Glycoproteins, such as extensins and arabinogalactan proteins (AGPs), as well as lignin have also been found in charophyte walls. Most charophytes also exhibit simple phenotypes when compared with those found in land plants. This simplicity has been exploited in many cell wall studies focused on fundamental phenomena, such as pectin dynamics and cell expansion (*Chara*) [22], as well as wall development during cellular morphogenesis (*Micrasterias*) [23,24]. Recently, my laboratory isolated a clone of the charophyte, *Penium margaritaceum,* and it has become increasingly valuable in cell wall studies. This alga is *unicellular*, produces only a primary cell wall that has clearly defined domains of cellulose, pectin and other wall polymers and can be easily manipulated for both microscopy-based analyses and experimental screening [25,26]. Recently, *Penium* was successfully transformed [27], and current efforts to sequence the genome are nearly complete. All of this contributes significantly to *Penium*’s present and future role as a model organism for cell wall studies. In this paper, the attributes of, and protocols for, using *Penium* in multiple areas of cell wall research are presented.

## 2. General Biology and Growth Characteristics

**Figure 1:**
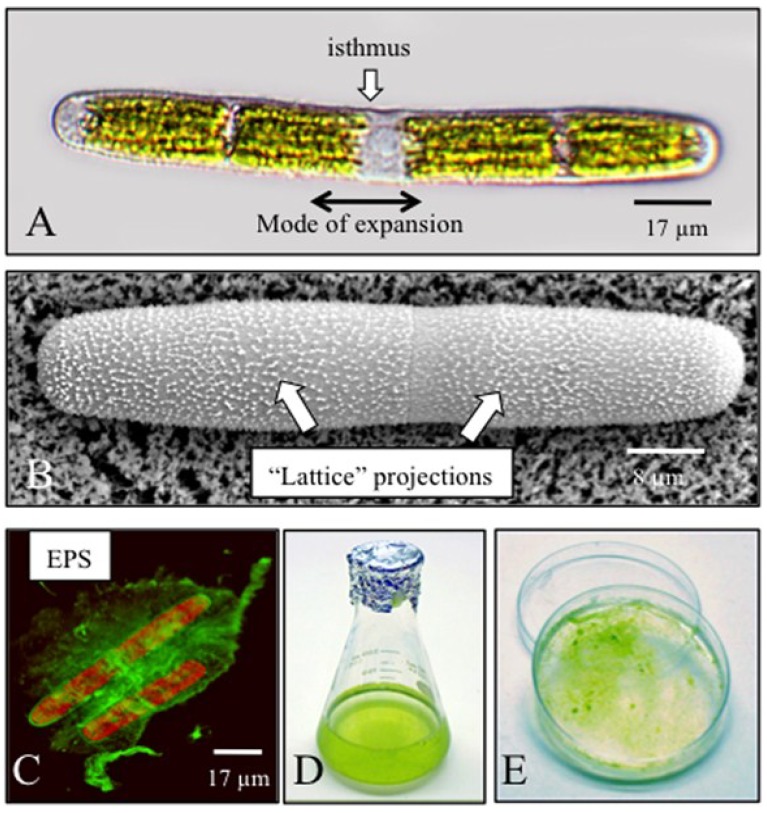
General features of *Penium* and its cell wall. (**A**) *Penium* is an elongated cylindrical uni-cell with rounded edges. The central isthmus (“isthmus”) contains the nucleus and is also the focal point of cell wall expansion. New wall polymers are deposited here in a narrow band that, in turn, displaces older cell wall outward toward the poles (“mode of expansion”, arrow); (**B**) variable pressure scanning electron microscopy (VPSEM) image of the cell wall surface showing the numerous projections that form the outer wall lattice (“lattice projections”, arrows); (**C**) *Penium* also produces a gel-like EPS that attaches to the wall and extends outward (arrow). The cell was labeled with an anti-EPS antibody and viewed with a confocal laser scanning light microscope (CLSM); (**D**) For culturing, cells are grown in flasks of sterile liquid medium; (**E**) cells may also be successfullygrown on agarose-solidified medium. In this image, the cells are growing on cellophane on top of Woods Hole medium (WHM) medium solidified with 1.5% agarose. EPS, extracellular polymeric substance.

*Penium margaritaceum* is a unicellular desmid whose shape is that of a simple elongate cylinder with rounded poles (Figure 1A). Each cell measures approximately 17 µm in diameter at the cell center or isthmus and attains lengths of 120 to 240 µm. A nucleus is positioned for most of the cell cycle at the isthmus and is surrounded by two elongated chloroplasts. Several different types of vacuoles are found throughout the cytoplasm, as are numerous crystals, most likely barium or silica salts, a feature that is common in desmids [28]. The peripheral cytoplasm is also the site of rapid cytoplasmic streaming and consists of narrow channels appressed to the plasma membrane that run approximately parallel to the long axis of the cell. The cell wall is approximately 1 µm thick throughout most of the cell surface [25]. The outermost cell wall layer is characterized by a distinct collection of projections or “warts” (Figure 1B) that forms a regular pattern. This layer has been designated as the pectin “lattice”. However, the wall is notably thinner and is devoid of the projections in a 1-µm band that encircles the isthmus [26]. Likewise, several narrow peripheral bands lined by different-sized projections are also found on the cell wall. The isthmus is the major site of the cell wall expansion mechanism, which begins prior to cell division [29]. Here, new wall components are deposited in a narrow band found at the center of the isthmus that, in turn, displaces older wall regions outward toward both poles (*i.e.*, “bipolar” expansion). Additionally, other areas of wall deposition have been noted in development [29]. During cell division, post-mitotic daughter nuclei are rapidly translocated from the isthmus, each to the center of a soon-to-be daughter cell. The exact mechanism for this event is not known for *Penium*, but is most likely microtubule-based, as noted for other desmids [30]. Cytokinesis occurs at the isthmus shortly after mitosis and entails both a cell plate and a small furrow. After cytokinetic separation, the poles of each daughter cell may expand for a short time thereafter. *Penium* also secretes a gel-like extracellular polymeric substance (EPS) beyond the cell wall [31]. The EPS is a xylose-, fucose- and glucuronic acid-rich polysaccharide(s) that is often produced in very large amounts, especially as the cells age or if the cells are exposed to high light, high temperature or desiccation conditions. The EPS may be imaged using immunofluorescence labeling of live cells (Figure 1C).

*Penium* cultures may be maintained in many types of algal freshwater media. My laboratory routinely uses liquid Woods Hole MBL (Marine Biology Laboratory) medium (WHM) [32], either alone or supplemented with 5% soil extract. Culture volumes of 75 to 150 mL in sterile 125- or 250-mL flasks are convenient and yield log phase cultures within seven days (Figure 1D). Subcultures are typically made every 10–14 days by transferring 5 mL of algal suspension to a new flask with fresh medium. Cells are also successfully grown on agarose-solidified WHM (1%–2% agarose), either directly on agarose-solidified medium or on sheets of sterile dialysis membrane laid on the surface of agarose-solidified medium (Figure 1E).

For harvesting cultures, cells grown in liquid culture are collected by gentle centrifugation at 500–1000 × *g* for 1 min in 15-mL or 50-mL centrifuge tubes. For washing and to remove EPS gel from the cells (EPS gel on the cell surface may alter subsequent labeling efficacy), post-centrifugation cell pellets are re-suspended in fresh growth medium, shaken vigorously for 10–15 s and then re-centrifuged. The supernatant containing EPS is discarded. This is repeated several times, and centrifugation results in tight green cell pellets free of EPS. These cells can then be used for cytochemical labeling, experimentation or cell wall isolation. For the production of large volumes of cells, cultures may be upscaled to 1-L volumes in 2-L flasks. If cultures become contaminated, washed cells may be treated with an antibiotic/antimycotic solution treatment (e.g., Sigma A5955; Sigma Chem., St. Louis, MO, USA; 1/100 dilution in WHM, overnight, followed by extensive washing in WHM).

## 3. The Cell Wall: Biochemistry, Labeling and Growth Monitoring

**Figure 2:**
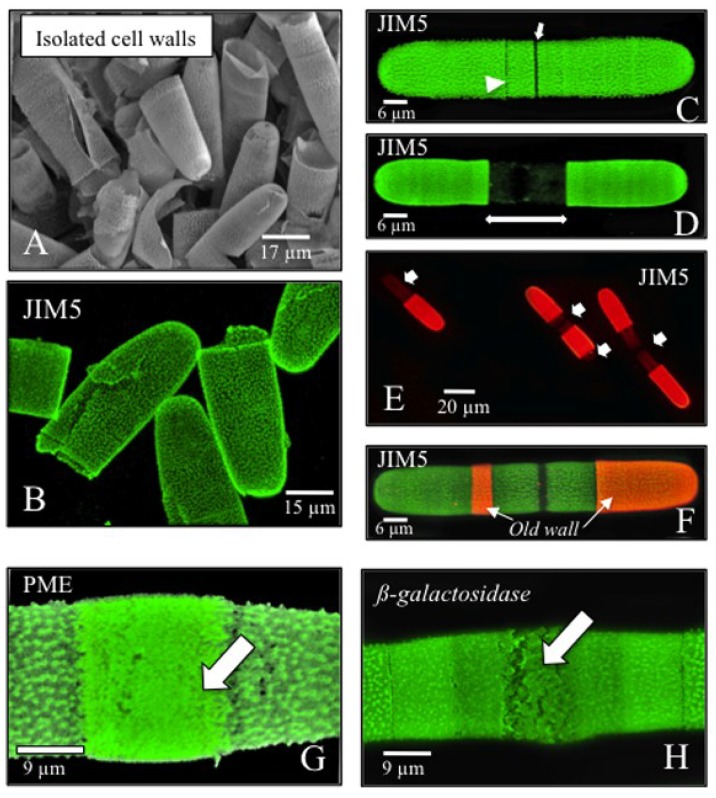
The cell wall and labeling with mAbs. (**A**) VPSEM image of isolated cell walls obtained through sonication (see text); (**B**) JIM5 immunofluorescence of the homogalacturonan (HG) of the outer wall layer of isolated cell walls. This mAb recognizes HG with relatively low levels of methyl-esterification. Note that the projections are clearly distinguished on the outer wall surface; (**C**) JIM5 immunofluorescence of a live cell. Most of the cell wall surface is labeled except for the isthmus (small arrow) and a lateral band (arrowhead); (**D**) After live labeling with JIM5, cells are placed back into culture. Over time, newly-deposited HG/cell wall appears as a dark zone centered at the isthmus (double arrow); (**E**) New growth zones and expansion rates in live labeled cells may be assessed using fluorescence microscopy. The surface areas of the dark zones (arrows) are measured and compared to overall cell surface area; (**F**) Live labeled cells that were allowed to grow in culture for 24 h and subsequently labeled with JIM5 that is, in turn, labeled with a different fluorophore reveals old (red) and new (green) wall regions. In this cell, the position and size of old wall regions (“old wall”, arrows) reveals an asymmetry in wall expansion [25]; (**G**) Live cell labeling with JIM5 is also used to assess alteration of the outer wall lattice. When treated with pectin methyl esterase (PME) for 24 h, the lattice at the isthmus is replaced by dense punctate labeling (arrow; see also [27]); (**H**) When cells are treated for 24 h with β-galactosidase, the lattice is disrupted (arrow).

The *Penium* cell wall consists of two dominant components, a homogalacturonan (HG)-rich outer layer that is complexed with calcium (Ca^2+^) and that is manifested by the lattice that is positioned on the cell wall surface [26]. The HG-rich outer layer is attached to an inner layer of cellulose microfibrils. Pure fractions of cell walls can be isolated using simple physical extraction techniques (Figure 2A) [25]. Log phase culture cells are collected by centrifugation, extensively washed and resuspended in an ice-cold buffered detergent solution (0.01% Triton-X-100 in 0.05 M Tris buffer, pH 7.2). The suspension is then sonicated on ice to cavitation for ten, five- to ten-s bursts. This procedure ruptures the cells and leaves the cell walls. The cell walls are collected by centrifugation, repeatedly washed with buffer and recentrifuged. This results in a pure white pellet. The purity of the pellet can be judged by light microscopy examination. If the pellet still contains whole cells, further sonication/washings are needed, and if starch grains are present, the pellet may be treated with amylase for a few hours and then washed. From pure cell wall pellets, various wall polymers can be extracted from the isolated walls using conventional cell wall extraction protocols (e.g., with chelators, base, acids or cadoxen). For example, the outer HG-rich layer is extracted from the isolated walls with 4-h incubations in 50 mM CDTA (1,2-Diaminocyclohexanetetraacetic acid monohydrate). The extract is then extensively dialyzed against deionized water and freeze dried for storage. Subsequently, when solutions of this pectin extract (e.g., 50–250 µg/mL in deionized water) are mixed with 10–25 mM solutions of exogenous Ca^2+^-salts or other cation-salts, transparent gels form rapidly, typical of HGs. The post-CDTA wall remnants may then be extracted with other agents, and the extracts are analyzed via biochemical/immunobinding protocols. It is important to note that prior to extraction, large amounts of EPS may be isolated from the medium of cell cultures or from the washes of cell pellets (see above). The EPS in these solutions may be concentrated by centrifugation at 25,000 × *g* for 10 min, where it forms a gel-like pellet. The EPS may be dialyzed against deionized water and freeze dried for storage prior to analysis.

A valuable feature of *Penium*’s cell wall is that it can be easily labeled via immunofluorescence in live cells or after removal from cells (Figure 2B; see [25] and [29] for detailed descriptions) using monoclonal antibodies (mAbs) currently available for the study of higher plant cell walls. For example, the HG-rich outer layer readily binds to mAbs specific for HGs derived from higher plants (e.g., JIM5, LM18, LM19, PAM1, 2F4, JIM7, LM20; [33,34,35,36,37]). Using a double labeling protocol, live cells may be labeled with a particular mAb and then labeled with the appropriate secondary antibody conjugated with a fluorophore (Figure 2C). The cells may be washed and then placed back into culture. Cells are not harmed by this process and continue to expand and divide at the same rate as unlabeled cells. The fluorescent label is very stable and typically maintains the same intensity level even after seven days. After a period of growth, new cell wall HG will appear as a dark zone situated between the mAb-labeled zones (Figure 2D,E). The surface areas of the dark zones may be measured to determine wall expansion rates during the cell cycle and under various experimental treatments. Likewise, after a designated period of time, the labeled cells may be labeled with the same mAb followed by labeling with a different secondary antibody-fluorophore conjugate. Imaging of the two fluorescent signals with different channels and/or filter sets of a confocal laser scanning light microscope (CLSM) allows for identification of old and new wall areas and growth patterns (Figure 2F). Similarly, another mAb with specificity for other wall polymer epitopes may be used in subsequent labeling to help reveal the precise location of different polymers in the wall and putative interpolymeric connections. For example, cells first labeled with JIM5, LM18, 2F4 (specific for relatively low methyl-esterified HG or Ca^2+^-complexed HG), re-cultured and then labeled with JIM7 or LM20 (specific for “relatively” high methyl-esterified HG) show that “high” methylesterified HG is secreted in a narrow band at the isthmus and is surrounded by wall loci with HG that has been de-esterified and complexed with Ca^2+^. Presumably, the enzyme, pectin methylesterase (PME), de-esterifies the HG at this band that, in turn, allows Ca^2+^ to cross-link to the HG and form the projections of the outer wall lattice. This live labeling technology is also valuable for studying the effects of specific experimental agents on the wall microarchitecture. For example, cells incubated in medium containing the enzyme, PME, for 24 h (Figure 2G) or β-galactosidase for 48 h (Figure 2H) and then labeled with JIM5 reveal significant alteration to the HG lattice at the isthmus (see also [26]). *Penium*’s “versatility” in immunocytochemical studies make it an excellent organism for screening new wall mAbs, as well as other probes, including carbohydrate binding modules (CBMs) [3], particularly those applicable for the analysis of cellulose and hemicellulosic polymers.

## 4. High Resolution Electron Microscopy Analyses

*Penium*’s unicellular habit also makes it a convenient model organism for electron microscopy-based studies of the wall dynamics, especially the secretory apparatus and cytoskeletal system. Earlier studies with other unicellular desmids, like *Micrasterias* [22,23], provided important insight into the development of protocols for cell preparation for *Penium*. For transmission electron microscopy (TEM), cells may be fixed with conventional glutaraldehyde/osmium tetroxide-based protocols, dehydrated, embedded in plastic and sectioned in order to provide good quality preservation of the cell wall. However, considerable shrinkage of the protoplast occurs via this method. However, all cell ultrastructure may be preserved satisfactorily using cryo-fixation and freeze substitution methodologies. For rapid cryo-fixation, a suspension of cells may be sprayed with a commercial artist’s spray brush and air compressor into liquid propane cooled in liquid nitrogen. This spray freezing is followed by freeze substitution in glutaraldehyde, osmium tetroxide and/or uranyl acetate in acetone or ethanol cooled to −80 °C for various periods of time. Cells are then warmed to either −20 °C or room temperature, washed with acetone or ethanol, infiltrated and embedded in plastic. We often employ osmium-free freeze substitution and London Resin embedding at −20 °C for cells to be subsequently sectioned and immunolabeled. For general ultrastructural examination, we use freeze substitution cocktails containing osmium and subsequently embed in Spurr’s low viscosity plastic at 60 °C. For both protocols, we find it convenient to place cells in a thin layer of pre-polymerized plastic sandwiched between two sheets of the Aclar plastic. After polymerization, the Aclar sheets are removed, individual cells of interest are selected with a light microscope and are excised from the sheet. The cells are positioned/glued onto a plastic block for sectioning at various orientations. It is also important to note that if sections of osmicated cells are first treated for 5 min in 5% H_2_O_2_, they can be successfully used for immunogold labeling with the wall polysaccharide-specific mAbs.

**Figure 3:**
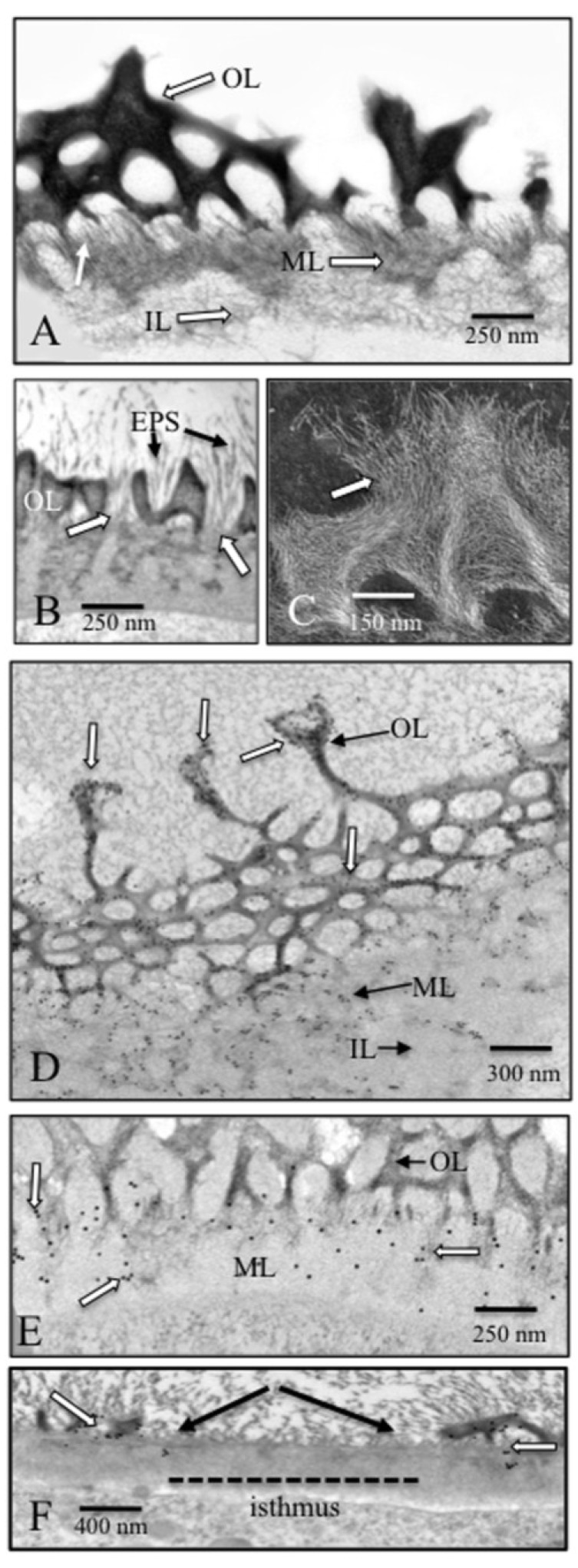
TEM imaging of the cell wall (see [25,27] for details). (**A**) Cross-section of the cell wall highlighting the distinctive outer layer (OL) that forms the lattice, the inner cellulose-based layer (IL) and the interface medial layer (ML). The medial layer is composed of fibrils from the outer layer that embed in the inner cellulose layer; (**B**) EPS fibrils (EPS) attach to the outer surface of the inner/medial wall (white arrows) and extend outward beyond the outer layer (OL); (**C**) Dark field image of the HG fibrils (arrow) that form dense aggregates that make the outer wall layer lattice. This cell was treated with a weak chelator to remove Ca^2+^ (see text); (**D**) JIM5 immunogold labeling (white arrows) of the HG-containing outer layer (OL) and medial layer (ML). The inner layer (IL) does not label with this mAb; (**E**) RG-I immunogold labeling (white arrows) of the medial layer (ML), but not the outer layer (OL); (**F**) The isthmus zone labeled with JIM5. Note the absence of the outer lattice in the isthmus (“isthmus”, arrows), but the presence of the lattice on the edges of the isthmus (white arrows).

TEM analyses reveal that the cell wall is approximately 1 µm wide throughout most of the cell, with the outer HG-lattice being 550 nm and the inner cellulose-based layer being 450 nm thick (Figure 3A). EPS fibrils attach to the outer portion of the inner wall layer and extend through the HG lattice and away from the cell surface for several µm (Figure 3B). The HG-rich lattice consists of projections made of struts that extend outward and fuse to form a single terminus. Mild EDTA extraction of cells or isolated cell walls in order to remove Ca^2+^ from the outer lattice reveals that the lattice struts consist of parallel arrays of hundreds of thin fibrils (Figure 3C). Fibrils from the inner portion of the lattice penetrate and embed in the inner cellulose microfibril-rich layer. This zone constitutes the medial wall layer (Figure 3D). Immunogold labeling shows that both the lattice and medial layer contain HG, while the medial layer alone contains HG and rhamnogalacturonan-I (RG-I; Figure 3E). This suggests that RG-I may serve as a linker between the HG and cellulose [27]. At the primary site of wall expansion, the isthmus, the cellulosic inner layer is the only wall layer observed and is devoid of the lattice. The medial layer and emerging outer lattice (Figure 3F) are found on either side of the isthmus region.

**Figure 4:**
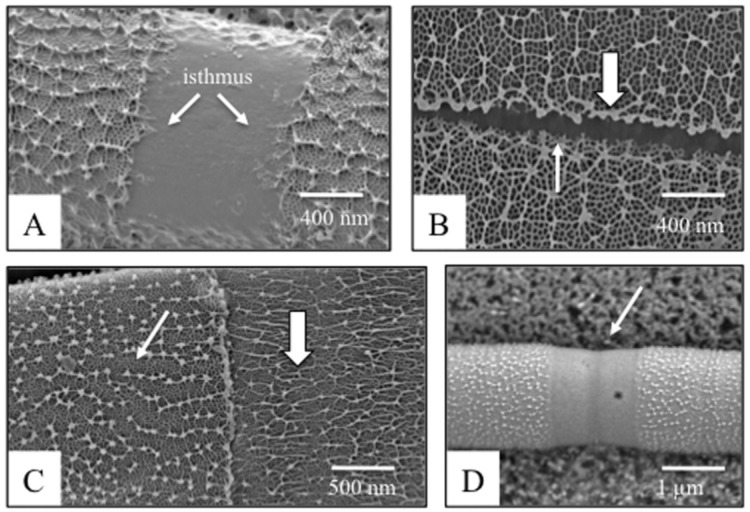
SEM imaging of the cell wall (see [25,27] for details). (**A**) FESEM image of the isthmus zone (“isthmus”, arrows). Note that the outer wall lattice projections are not present in the isthmus; (**B**) FESEM image of a lateral band. On the outer edge of the band (*i.e.*, facing a pole of the cell), the projections are thick (large arrow). On the inner edge, the lattice is smaller (small arrow). No projections are found in the middle of the band; (**C**) FESEM image of a cell incubated for 48 h in medium supplemented with 10 × CaCl_2_. The older wall (small arrow) possesses the typical outer lattice. However, in the newer wall zone formed when under high Ca^2+^ conditions, the lattice forms a series of elongated ridges (large arrow; see also [27]); (**D**) VPSEM image of a cell treated for 48 h with PME. Note that at the isthmus, *i.e.*, the focal point of wall expansion, the outer lattice is missing (arrow).

The ultrastructure of the *Penium* wall may also be analyzed using scanning electron microscopy (SEM). Field emission scanning electron microscopy (FESEM) of both frozen and isolated cell walls clearly shows the lack of the lattice at the isthmus (Figure 4A), but also reveals narrow lateral bands found at more polar regions of the cell (Figure 4B). Close examination shows that the lattice projections are taller on the pole-facing side of these lateral bands than on the isthmus-facing side. The role of these bands is unknown, but may very well represent areas of growth oscillations. For example, wall synthesis does not occur during the dark period of the photocycle. It is may be that the lattice formation stops abruptly when the cell is placed in the dark, resulting in normal size projections. However, when returned to the light, the lattice formation mechanism is more gradual, and those projections are notably smaller. FESEM also has been important in revealing that the arrangement of cellulose microfibrils on the innermost surface of the cell’s isthmus runs perpendicular to long axis of the cell at the isthmus [27]. FESEM is also used to identify changes to the HG lattice when cells are treated with agents that affect HG secretion and incorporation into the wall. For example, in cells grown in cultures containing 10 times the normal concentration of CaCl_2_, large elongated ridges replace the individual projections of the typical lattice (Figure 4C).

Though not as powerful a high resolution imaging tool as FESEM, variable pressure scanning electron microscopy (VPSEM) allows for rapid assessment of wall structure. Here, cells treated in various ways can be collected and placed on nitrocellulose sheets. The sheets are plunge frozen in liquid nitrogen and secured upon on a cooled stage that is placed in the VPSEM. The vacuum pressure of the SEM column is brought to 20–30 Pascal, and within minutes, sublimation removes the ice from the cell surface, to reveal structural changes (Figure 4D). This technology has proven to be exceptionally helpful in quickly screening cells treated with a variety of experimental agents.

## 5. Experimental Analyses: Microarray or Single-Cell Approach

*Penium*’s unicellular cylindrical shape and distinct cell wall along with a fast growth rate provide an outstanding model system for the screening of large numbers of experimental agents and gauging their effects on cell and wall structure and development. Cells incubated in medium containing various agents and concentrations thereof may be grown in 12-, 24- or 48-welled petri dishes. Typically, concentrations of 1000 cells/mL in various aliquots are used per well. The cells may also be first labeled with a wall polymer-specific mAb-fluorophore (see above) before treatment. The effects of the agent may then be conveniently viewed using an inverted light or CLSM. For cells initially labeled with a wall-specific mAb, growth rates, as identified by the surface area of non-labeled zones *vs.* whole cell surface area, may be measured. Aliquots of cells from individual wells may also be harvested and labeled for immunofluorescence or processed for electron microscopy. Likewise, aliquots of treated cells from each well may be harvested, washed and placed in fresh growth medium in order to monitor recovery dynamics after an experimental treatment. Finally, individual cells of *Penium* may be isolated using sterile tips of a 0.5–20 µL micropipettor and subsequently grown in medium contained in wells of a 96-welled petri dish. This allows for imaging individual cell histories over time and the monitoring of individual cell dynamics under specific experimental treatments. These protocols have been especially valuable in studies of wall development in cells treated with cytoskeletal poisons, various cations, enzymes and exogenous pectins [26,38,39].

## 6. Intracellular Studies

**Figure 5:**
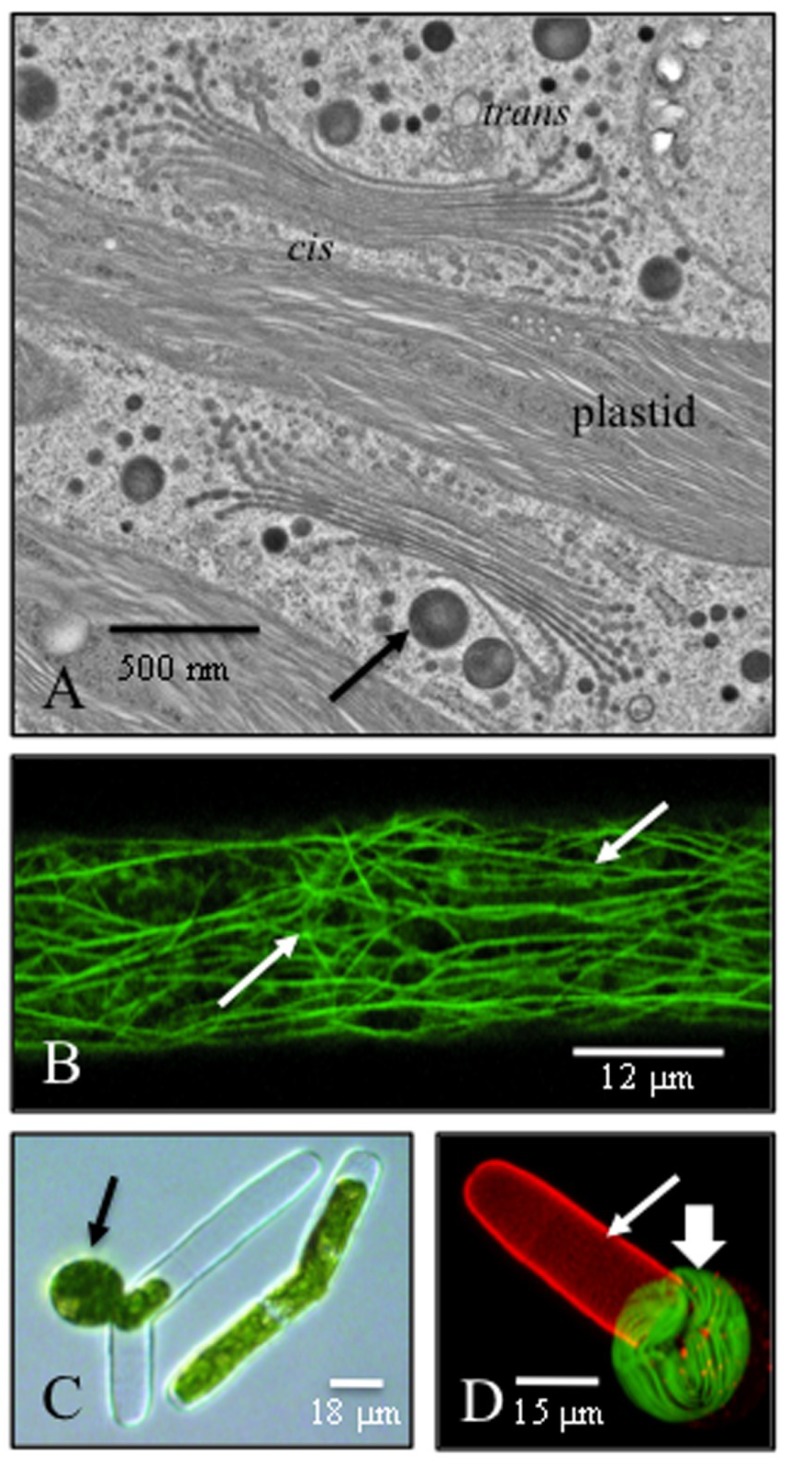
The cell and protoplasts (see [39,40,43] for details). (**A**) A TEM image of two of the 100–125 Golgi bodies of the cell. The Golgi bodies sit at the base of lobes of cytoplasm lined by lobes of the chloroplast. Clearly defined cis (*cis*) and trans (*trans*) faces are apparent. Vesicles emerge from the trans of the medial loci of the Golgi body (arrow) and travel to the peripheral cytoplasm, where they enter the peripheral cytoplasmic streaming network; (**B**) Rhodamine phalloidin labeling of the actin bundles in the peripheral cytoplasm (arrows); (**C**) Protoplast release through the cell wall at the isthmus zone (arrow). The cell was treated with Driselase in a 12% mannitol solution (work to be reported elsewhere); (**D**) Fluorescence image of protoplast (large arrow) emerging from the cell wall (small arrow) labeled with the mAb, JIM5.

*Penium* contains an elaborate endomembrane system consisting of 100–125 Golgi bodies per cell, numerous vesicle types involved in secretory activities and a highly dynamic cytoplasmic streaming network that transports the vesicles. These subcellular components are involved in the biosynthesis and secretion of the cell wall and EPS, but many of the specific details have not yet been resolved. The Golgi bodies of *Penium* do not move notable distances in the cell, but remain stationary in the bases of valleys of cytoplasm lined by lobes of the chloroplasts. This is unlike what has been described for higher plants, whereby Golgi bodies are mobile [40,41]. Each Golgi body of *Penium* contains 12–16 tightly stacked cisternae and displays distinct *cis* and *trans* faces (Figure 5A) [42]. Large EPS-containing vesicles emerge from the peripheries of *trans* face cisternae, while wall polymers, like pectin, appear to be packaged in small vesicles arising from a more medial region of the Golgi body. All vesicles move to the cell periphery where they become part of the active cytoplasmic streaming network. This streaming mechanism is associated with bundles of actin cables that may be imaged using rhodamine phalloidin-based labeling protocols (Figure 5B) [43] or TEM. *Penium*’s microtubule network may also be imaged using freeze shattering and labeling with anti-tubulin [40]. The isthmus zone is almost always lined with a band of microtubules aligned perpendicular to the long axis of the cell. These microtubules are likely associated with wall synthesis in this area of active wall expansion, most likely as agents participating in cellulose microfibril biosynthesis and/or as directional targets for vesicle-plasma membrane fusion. This band also marks the future site of mitosis and cytokinesis, *i.e.*, very much like a preprophase band found in embryophytes [44,45]. Interestingly, when the cell starts to expand prior to cell division, satellite bands arise on either side of the isthmus band and migrate toward the poles of the cell during expansion. These bands represent the sites of future cell division and wall expansion, in effect pre-prophase bands for the next division phase [40]. Further elucidation of these processes using transformed cell lines will certainly aid in understanding the dynamic activities associated with these subcellular systems.

## 7. Selective Dissolution of the Cell Wall and Protoplast Formation

Protoplasts of plant cells are important tools for experimentation (e.g., transformation, biochemical syntheses) and helping to resolve key questions in plant cell biology and biochemistry [46,47,48]. They also provide a unique system for monitoring recovery dynamics of the cell wall after it has been removed from the cell. Protoplasts may be isolated from *Penium* when log phase cells are incubated in a mannitol-based osmoticum containing 1% Driselase (Figure 5C,D). Typically, within 20 min, high yields of protoplasts (80% or more of the cells yield protoplasts) are released from the altered cell wall and may be cultured (work to be reported elsewhere). Though viable for several days in osmoticum, the recovery dynamics of the cell wall have yet to be described. As important, live *Penium* may also be incubated in medium containing specific wall-degrading enzymes. Specific polymers of the wall are selectively removed, and the architecture of the altered wall are monitored by immunofluorescence or electron microscopy analyses. Recovery dynamics may also be observed in washed cells that are returned to culture. Here, the isthmus zone serves as the initiation site of recovery [26]. These enzymatic dissections of the cell wall in live cells represent a powerful means of assessing the role of particular polymers in cell wall architecture, cell shape maintenance and cell expansion.

## 8. Molecular Biology: Transformation Technology

Surprisingly, there is a paucity of both detailed genomic sequencing and annotation in the charophytes today [49,50]. This has hindered our understanding of evolutionary trends and biosynthetic/metabolic pathways associated with the cell wall in these organisms. Recently, though, a major breakthrough occurred when *Penium* was successfully transformed [27]. *Agrobacterium*-based transformation was accomplished with cells initially incubated in nitrogen-depleted medium. This allowed for the establishment of cell lines displaying stable RFP-KDEL expression and provided novel insight into the network of endoplasmic reticulum in the cell. Likewise, RNAi-mediated gene suppression was used for the first time to elucidate PME and cellulose synthase (CESA). This exciting discovery and new technology offers a powerful and non-invasive means of studying specific proteins in *Penium*. When coupled with future genomic sequencing information, elucidation of *Penium*’s cell wall dynamics will be enhanced significantly.

## 9. The Future

The first decade of detailed studies of *Penium* have yielded results that demonstrate the importance of this unicellular organism as a model organism for studying plant cell walls. Its versatility in analysis using multiple and diverse technologies gives plant cell wall biologists and chemists a convenient and easily manipulable organism for obtaining insight into the fundamentals of cell wall structure and function. Some of the next major steps that need to be undertaken with *Penium* include: (1) analyzing the elaborate endomembrane system in order to identify the locations of key biosynthetic and secretory events associated with wall development; (2) identifying the pathways of endocytosis and its role in regulating expansion and wall polymer retrieval; (3) performing detailed biochemical dissections of the cell wall in order to determine the role of RG-I in HG-cellulose interactions and the location/functions of wall glycoproteins, also components of the wall; and (4) elucidating the chemistry and secretory dynamics of EPS, as it represents a major source of extracellular polysaccharide associated with the cell wall. Clearly, the use of unicellular charophytes, like *Penium*, provide new opportunities to resolve many questions about the cell wall that cannot be addressed in multicellular organisms.
